# A Rare Case of Dual Cystic Ducts

**DOI:** 10.7759/cureus.111878

**Published:** 2026-07-01

**Authors:** Heath P Darrant, Mia Langnier, Nestor Sabat, Sheila Fitzgerald

**Affiliations:** 1 Medical Education, University of New South Wales, Wagga Wagga, AUS; 2 General Surgery, Wagga Wagga Base Hospital, Wagga Wagga, AUS

**Keywords:** aberrant cystic duct, cholecystectomy, dual cystic duct, intra-operative cholangiogram, laparoscopic cholecystectomy

## Abstract

This case describes a rare dual cystic duct with low medial insertion identified during an elective laparoscopic cholecystectomy in a 49-year-old woman. Intraoperative difficulty arose when anatomical variation resulted in difficulty confirming Strasburg’s Critical View of Safety (CVS).

With the assistance of a cholangiogram and upper gastrointestinal surgical consultation, the patient underwent a dual duct ligation and an uncomplicated cholecystectomy. This case highlights the surgical complexity of cystic duct variants, reinforces the necessity of achieving the CVS, and underscores the role of ongoing surgical education.

## Introduction

The biliary tree typically originates as the right hepatic duct (formed by the anterior and posterior branches) and the left hepatic duct (LHD), which unite to form the common hepatic duct (CHD). This Type 1 configuration is described by the Huang classification. The cystic duct, arising from the gallbladder, usually inserts laterally at the CHD midpoint, forming the common bile duct (CBD) [1,2].

Cadaver, imaging, and intra-operative assessments of the biliary tree show broad anatomical variation on a population level. Around 20-40% of adults demonstrate at least one biliary variation, commonly involving the right and left hepatic duct junction [1,2]. The most common (11-21%) is Type A3, where the right posterior hepatic duct (RPHD) or right anterior hepatic duct (RAHD) inserts into the LHD. Type A2 (11-14%) describes a common junction of RPHD, RAHD and LHD, while Type A4 (5-10%) occurs when RPHD or RAHD merges directly with the CHD [1].

Cystic duct variation occurs in 18-30% of patients [3,4]. Medial insertion is most frequent (2.6-16%), followed by low insertion (1-9%) and high insertion (0.36-6%) [5-8]. Patients with anatomical variability experience increased complication rates, such as a 2.4-fold increase in biliary leaks, haemorrhage rates 9.8 times higher and higher incidences of laparoscopic to open conversion [9]. Reinforcing the importance of a systematic approach, such as Strasberg's Critical View of Safety (CVS). 

The CVS is designed to allow surgeons to visualise key anatomy such as the cystic duct and cystic artery, whose injury can be life-threatening. The CVS is achieved by dissecting the lower third of the gallbladder and clearing fibrous/connective tissue from Calot’s triangle. Removal of fibrous tissues exposes the cystic duct and artery, allowing for surgical identification, mapping and operation planning, thereby reducing surgical complications [10].

## Case presentation

A 49-year-old woman underwent laparoscopic cholecystectomy following one episode of cholecystitis and two episodes of biliary colic over nine months. CT at first presentation showed acute cholecystitis, choledocholithiasis and CBD dilation (Figure 1). Ultrasound confirmed multiple gallstones, with two impacted in the neck (Figure 2). She was treated with antibiotics and supportive care, recovering well without surgical intervention. The subsequent episodes of biliary colic were self-resolving without complication or the need for urgent surgery. Thus, she underwent elective cholecystectomy nine months after her initial presentation.

**Figure 1:**
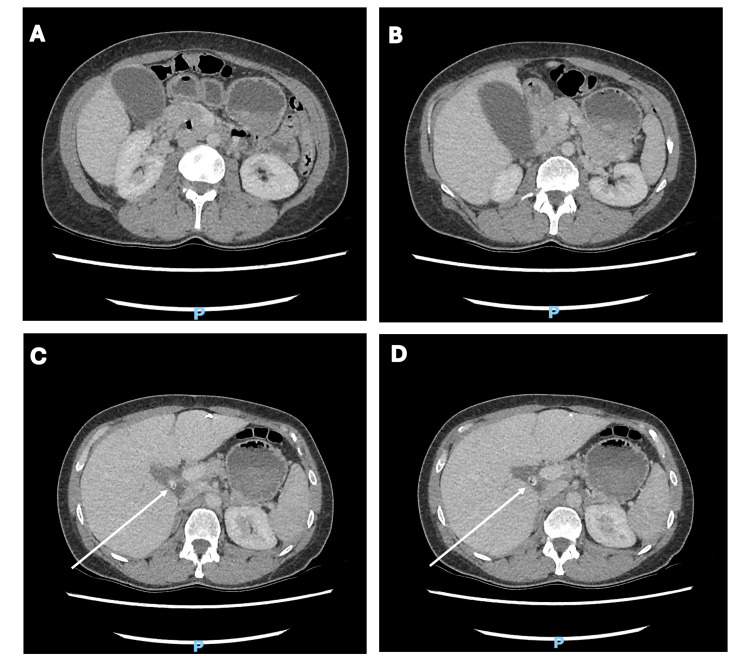
CT at initial presentation demonstrated evidence of cholecystitis with associated choledocolithiasis. This panel series, moving caudal to cranial through from A to D, shows the extent of gallbladder dilation and wall thickening. The presence of gallstones within the cystic duct and superior aspect of the biliary tree can also be observed, marked by the white arrows in films C and D.

**Figure 2:**
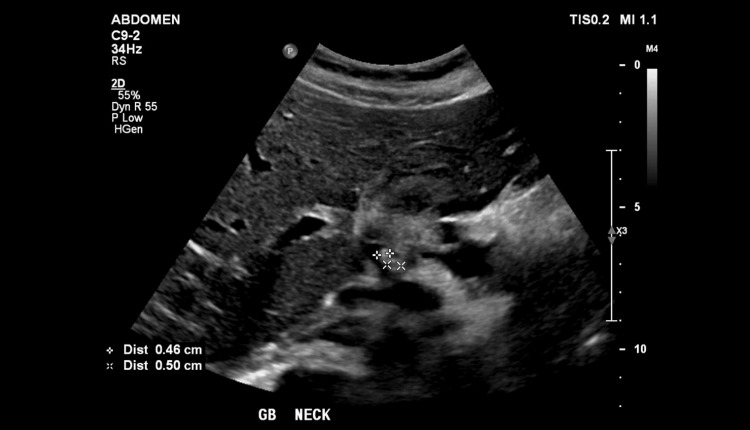
The ultrasound still image shows two stones impacted within the gallbladder neck. Both stones are of comparable size, 0.46cm and 0.50cm, respectively.

Intraoperatively, the CVS was established following liver retraction. Upon clearance of fatty tissue, aberrant anatomy was identified, specifically, a dual cystic duct. Immediately, intraoperative cholangiogram (IOC) was organised, confirming a low cystic duct insertion into the CBD and a second duct connecting the right hepatic duct to the cystic duct, as seen in Figure 3. No CBD filling defects were noted. With upper GI surgical consultation, both cystic and aberrant ducts were clipped and resected. The cystic artery was then ligated and the gallbladder was removed without complication.

**Figure 3:**
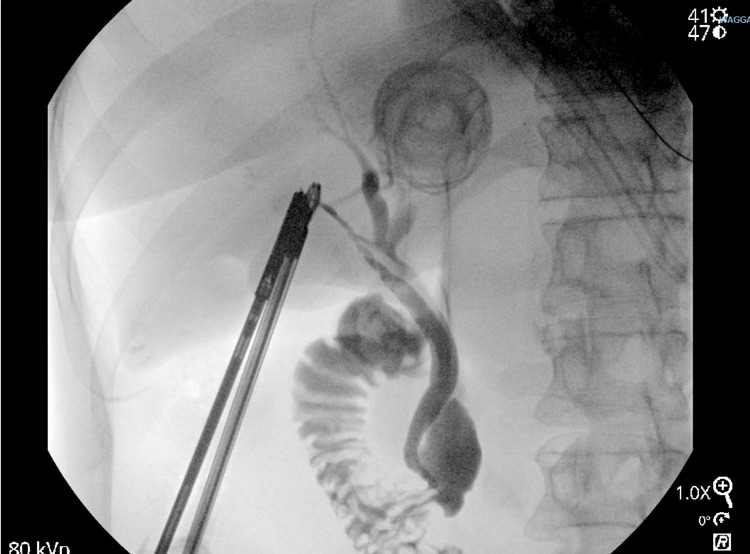
Intraoperative cholangiogram performed prior to cystic duct clipping. The image demonstrates a bifurcated cystic duct. The major branch continues inferiorly and inserts into the lower third of the common bile duct. The aberrant duct however courses superiorly to join at the level of merger between the right and left hepatics, forming the common bile duct.

A distal dilatation seen on cholangiogram raised suspicion for a choledochal cyst. Follow-up magnetic resonance cholangiopancreatography (MRCP) four weeks postoperatively, refer to Figure 4, instead revealed an extraluminal duodenal diverticulum.

**Figure 4:**
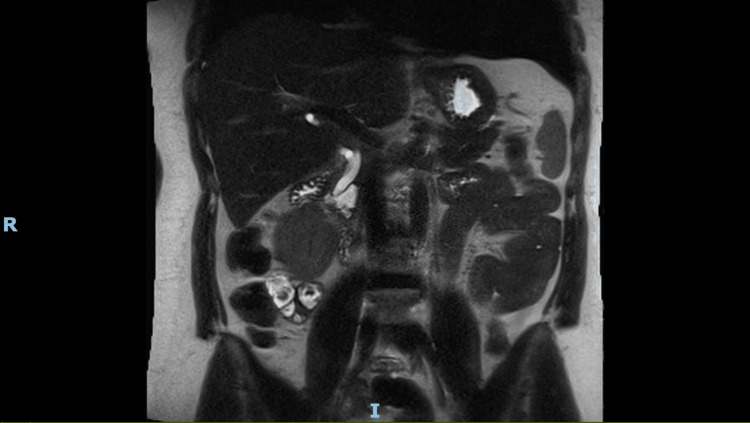
As observed in Figure 3, there is a dilatation medial to the base of the biliary tree. The MRCP still image assists in classifying this dilatation as a duodenal diverticulum, specifically at the second part of the duodenum. MRCP: Magnetic resonance cholangiopancreatography

## Discussion

This case highlights the importance of appreciating anatomical variation within the biliary tree and its potential to complicate standard surgical procedures such as laparoscopic cholecystectomy. As observed in Figure 3, this patient demonstrates a rare combination of cystic duct anomalies: a low medial insertion and a dual (accessory) cystic duct. A simplified schematic of this layout can be observed in Figure 5. Both of these abnormalities are extremely uncommon and pose a significant risk for inadvertent injury [6,11]. As of 2025 less than 15 case reports document dual cystic ducts [12-14]. Additionally, up to 80% of such cases are associated with a dual gallbladder, highlighting the rarity of this patient’s anatomy [12,14].

**Figure 5:**
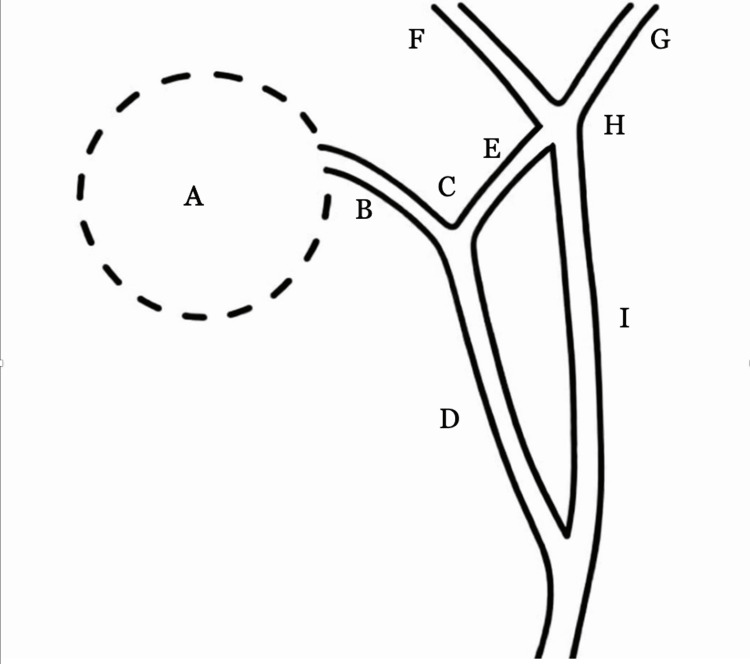
Diagram depicting the aberrant bifurcation of the cystic duct. The cystic duct (B) leaves the gallbladder (A) and bifurcates (C) into an aberrant (E) and major duct (D). The major branch joins the lower third of the common bile duct (I) inferiorly, whilst the aberrant branch merges superiorly at the junction between left (G) and right (F) hepatic ducts. A. Excised gallbladder B. Cystic duct C. Bifurcation of the cystic duct D. Major branch of the cystic duct E. Aberrant cystic duct F. Right hepatic duct G. Left hepatic duct H. Merger of right and left hepatic ducts I. Common bile duct Figure created by Mia Langnier using Notability

Anatomical variance unfortunately does not present with a unique clinical presentation or cluster of biliary symptoms. Therefore, recognition of variants pre- or intra-operatively through dissection and cholangiography is crucial. Misidentification, particularly with inflammation, adhesions, or distorted anatomy, can cause severe complications such as CBD transection or strictures [15]. Bile leaks occur in 15.7% of these patients versus 6.4% with normal anatomy. Haemorrhage is reported at 16.8% compared to 1.9% [9].

Additionally, the implementation of IOC further stratified the patient anatomy, raising concerns for a choledochal cyst, a congenital anomaly that predisposes patients to cholangitis, biliary strictures, and malignancy, in addition to stone formation [16]. Abnormal ductal dilation contributes to bile stasis and increases the risk of de novo choledocholithiasis, particularly in the absence of gallbladder drainage and altered biliary pressures post-cholecystectomy.

In this context, the risk of recurrent biliary obstruction, ascending cholangitis, and malignancy remains elevated. Although no stones were identified intra-operatively, the presence of a choledochal cyst would warrant long-term surveillance. Even more likely to occur, strong evidence now recommends elective surgical correction of most choledochal cysts, typically via complete excision of the dilated segment with biliary-enteric reconstruction (e.g., Roux-en-Y hepaticojejunostomy), to prevent future complications [16,17]. Fortunately for the patient, MRI revealed the presence of a duodenal diverticulum, asymptomatic in 95% of cases, with no intervention required [18]. Although there was an estimated prevalence rate of 22%, duodenal diverticula are not without their risks. Such a diverticulum can be complicated by ulceration, pancreatitis, cholangitis, intestinal obstruction and perforation [19]. Therefore, in-depth patient education, symptom safety netting and clear medical documentation are required to ensure prompt and accurate medical management if required in the future, thus, highlighting the critical role of intra-operative cholangiography in preventing iatrogenic injury in complex biliary anatomy and for postoperative planning when incidental findings are identified.

## Conclusions

This case report is a primary example of the surgical complexities that arise in the setting of anatomical variance. To minimise risk, surgeons should use multiple safety checkpoints, with Strasberg’s CVS a cornerstone of safe cholecystectomy. Similarly, IOC provides valuable clarity for guiding surgical approaches. Given the frequency of variation, surgeons must operate systematically yet remain adaptable when barriers arise.
